# Assessing the variability and vulnerability of carbon function in coastal soft sediment ecosystems to inform protection

**DOI:** 10.1002/eap.70234

**Published:** 2026-04-23

**Authors:** Tegan Evans, Ines Bartl, Shahrokh Heidari, Rebecca V. Gladstone‐Gallagher, Stefano Schenone, Patrice Delmas, Simon F. Thrush

**Affiliations:** ^1^ Institute of Marine Science Waipapa Taumata Rau Auckland New Zealand; ^2^ School of Computer Science Waipapa Taumata Rau, The University of Auckland Auckland New Zealand

**Keywords:** biodiversity recovery, blue carbon, carbon cycling, coastal ecosystems, conservation, demersal fishing impacts, fishing impacts, marine protected area, microtopographic surface features, sediment resuspension, soft sediment ecosystems

## Abstract

Coastal soft sediment ecosystems can sequester and process large quantities of carbon, making these environments important in mitigating the impacts of climate change. However, demersal fishing methods can resuspend sediment, releasing CO_2_, and in the longer term change the physical, biological, and biogeochemical characteristics of the seafloor. Within the global response to climate change, there is a need for tools to support the identification of sites within our coastal zones that can maximize carbon storage and where marine protection will be most beneficial for both blue carbon budgets and biodiversity recovery. We assess multiple aspects of carbon cycling in coastal soft sediment environments to explore the drivers of function and their vulnerability to seafloor disturbance. This information is interpreted to demonstrate how it can support decision‐making for enhancing blue carbon and marine protection for climate change mitigation. We found carbon cycle functionality is driven across multiple gradients, and sites with large infauna were more vulnerable to resuspension‐induced CO_2_ release. The results demonstrate that by accounting for environmental variability and complexity in marine protection, we have the opportunity to enhance multiple aspects of carbon cycling, protect biodiversity, and manage the vulnerability of sites to resuspension‐induced CO_2_ release.

## INTRODUCTION

The ocean plays an important role in global carbon cycling and is increasingly being recognized as part of the solution to managing the impacts of climate change (Macreadie et al., 2017; Roberts et al., 2017; Sala et al., 2021; Solan et al., 2020). Some ecosystems are natural carbon sinks, and the carbon that is captured within the marine environment is referred to as blue carbon (Nellemann & Corcoran, 2009). The role of coastal plants such as seagrass, mangroves, and saltmarshes has typically been the focus of blue carbon research (Duarte et al., 2013; Hilmi et al., 2021; Macreadie et al., 2019); however, these ecosystems only cover approximately 0.2% of ocean area, with the vast majority of the ocean being soft sediments (Snelgrove, 1999). Ocean sediments represent a vital carbon sink, storing large quantities of global carbon stocks (Atwood et al., 2020). Marine carbon dioxide removal methods are being explored to address the climate crisis but require caution and careful consideration of the ecological implications of such methods (Nawaz & Lezaun, 2024). Restoring and enhancing blue carbon environments through marine protection, however, does not carry the uncertainty of geoengineering methods and can also enhance other critical ecosystem services such as increased water quality, links to cultural well‐being, and biodiversity conservation. Despite the vast coverage and importance of sedimentary environments, they are often impacted by anthropogenic activities, particularly by demersal fishing. Trawling and dredging disrupt not only the physical, biological, and biogeochemical characteristics of the seafloor (Oberle, Storlazzi, & Hanebuth, 2016), but the resuspension of sediment releases CO_2_ (Sala et al., 2021). Marine protection could be used to mitigate this human impact on carbon stocks by prohibiting the use of demersal fishing gear and other extractive activities. To support informed decision‐making for marine protection, we need to understand the key drivers of variability in carbon cycle functionality across the heterogeneous seafloor. Understanding how the carbon cycle varies over environmental gradients allows us to identify areas within coastal zones that can maximize carbon cycle functionality and where marine protection will be most beneficial for blue carbon budgets.

While marine protection is often centered around enhancing biodiversity, it also has the potential to support the response to climate change (Elsler et al., 2022; Nellemann et al., 2009; Roberts et al., 2017; Sala et al., 2021). High levels of marine protection that exclude dredging and trawling are expected to have the greatest benefits for restoring biodiversity and adapting to climate change (Grorud‐Colvert et al., 2021), highlighting the need for both to be a focus for conservation efforts (Elsler et al., 2022). Demersal fishing methods have been estimated to release approximately 0.34–0.37 Pg CO_2_ year^−1^ into the atmosphere each year, which could add an additional 0.2–0.5 ppm to atmospheric CO_2_ concentrations by 2030 (Atwood et al., 2024). In addition to the need to reduce carbon emissions, protecting the current stores of carbon is critical. Atwood et al. (2020) estimated marine sediments contain approximately 1.75 times more carbon than terrestrial soil, with 48.8% of that being contained within Exclusive Economic Zones (EEZ). While decision‐making on implementing marine protection could be managed within national frameworks for almost half of the sedimentary carbon stocks, only approximately 2% are stored in fully or high protected areas (Atwood et al., 2020), which highlights the currently underutilized capacity of marine sediments as a blue carbon source that could support climate change mitigation efforts.

Marine sediments can both capture and store carbon through a series of ecosystem functions, making them critical in the response to climate change (Shin et al., 2022). Given their proximity to land, coastal environments in particular have a carbon cycle that is strongly influenced by the environment as well as anthropogenic activity. CO_2_ that is released by respiration and as a product of anthropogenic activity can be captured by microphytobenthos for photosynthesis, which converts CO_2_ into organic matter. Benthic primary production forms the base of the food chain on the sunlit seafloor, and once ingested, this carbon is stored as living tissue in benthic and pelagic fauna as secondary production. Microphytes within the sediment can influence how organic matter is broken down for storage (Canfield, 1994). Not all carbon is ingested or stored in plant and animal tissue, and some will be returned to the seafloor as feces, pseudofeces, or dead tissue. Benthic macrofauna assist in moving carbon through the environment, including filter feeders bringing organic matter from the water column to the seafloor and deposit feeders consuming and moving organic matter within the seafloor (Snelgrove et al., 2018). If left undisturbed, some of this organic carbon can be transferred into long‐term carbon storage deeper within the sediment. If disturbed, resuspending sediment can induce carbon remineralization, resulting in CO_2_ being released. Disturbance can originate from biological and physical sources. Biological sources include benthic fauna bioturbating and excavating sediment (Lohrer et al., 2004; Widdicombe et al., 2000) and megafaunal feeding such as rays moving large amounts of sediment (Vallim et al., 2024). Physical processes can include wave energy at the seafloor. Humans add an additional element of disturbance to the seafloor through activities such as anchoring (Watson et al., 2022) and demersal fishing (Atwood et al., 2024). CO_2_ is also released through the calcification process (Zhou et al., 2024). Ultimately, carbon cycling in marine sediments is complex and is influenced by the varying conditions of that environment. Disturbance of the seafloor can disrupt these conditions, which impacts how the system cycles carbon.

Demersal fishing methods change benthic community composition (Collie et al., 1997; Dayton et al., 1995; de Juan et al., 2011; McLaverty et al., 2021; Queirós et al., 2006; Thrush et al., 1998; Tillin et al., 2006), resuspend sediment (Oberle, Storlazzi, & Hanebuth, 2016), and change the physical and biogeochemical properties of the seafloor (Oberle, Swarzenski, et al., 2016; Tiano et al., 2019), which can have a number of impacts on functionality. Large benthic fauna play an important role in bioturbating sediment and connecting the water column to the seafloor, which can both move organic carbon deeper into the sediment for storage and make the surface sediment more nutrient rich to support microphytobenthos (Thrush & Dayton, 2002). Based on their life history traits, large fauna are generally more vulnerable to dredging and trawling, so are often removed from the ecosystem, reducing this functionality (McLaverty et al., 2021; Queirós et al., 2006). The resuspension of sediment can increase turbidity in the overlying water column for up to 5 days after the fishing event (Bradshaw et al., 2021; Palanques et al., 2001). Resuspending sediment can change the grain size of sediment as fine fractions are winnowed away (Martín et al., 2014; Oberle, Swarzenski, et al., 2016; Palanques et al., 2014), and shells are broken up and damaged (Ramsay et al., 1998), as well as decreasing the photosynthetic capacity associated with low incident light at the seafloor (Tiano et al., 2024). Resuspension can cause enhanced remineralization of organic carbon that could have otherwise been stored, which can release CO_2_ into the water column, leading to ocean acidification and add to the excess of CO_2_ in the atmosphere (Epstein et al., 2022; Sala et al., 2021). These varied responses to demersal fishing on soft sediment ecosystems highlight the need to understand the drivers of carbon‐related functions to inform how they can be adequately managed.

The implementation of meaningful marine protection requires place‐based data to support site selection (Pressey et al., 2015). Single abiotic variables such as grain size or water depth are often used in seafloor habitat classifications, which lack consideration for how diversity and ecosystem function vary across the landscape (Reiss et al., 2015). The lack of primary data in the management of soft sediment environments can in part be linked back to the challenges of collecting data in the marine environment, as well as data not being collected at a scale that is relevant to management (Clinton et al., 2024). As a result, there is a need to develop methods that allow for cost‐effective assessments that are informative for management and can capture variability at relevant scales. In recent years, there have been exciting advances in, for example, photogrammetry and video quantification of surface features that have been linked to measures of biodiversity and function (e.g., Azhar et al., 2022; Schenone, Bartl, & Thrush, 2024; Schenone, Hewitt, et al., 2024; Schenone & Thrush, 2020). Data collected using cameras, compared to traditional methods of grabs or cores, can save time and resources in both the data collection and processing phases, as well as support the collection of data over larger areas. Blue carbon assessments are often focused on vegetated habitats (Macreadie et al., 2017) or focused on first‐order measurements of CO_2_ production (Epstein et al., 2022), presenting a gap in how assessments can be applied in soft sediment habitats (Graves et al., 2022). The development of assays that can assess CO_2_ production across the heterogeneous seafloor (e.g., Bartl et al., 2024) advances the way that we are able to assess blue carbon in soft sediment environments. These methods that generate place‐based information of diversity and function over larger scales present opportunities for meaningful decision‐making.

If marine protection is to be used to aid the global response to climate change, decisions on where to protect need to include consideration of the multiple ecosystem functions that underpin carbon cycling (Evans & Thrush, 2023). The simultaneous assessment of multiple functions (termed multifunctionality) is critical to ensure protection does not focus on maximizing one function (e.g., carbon burial) to the detriment of others (e.g., primary and secondary production). There are a variety of approaches for assessing multifunctionality that may be useful in assessing carbon cycle functionality on the seafloor (Manning et al., 2018), including the development of single multifunctionality indices (Byrnes et al., 2014). There is a need for ecological indices to be transparent and comprehendible to users (Jørgensen et al., 2013), so the convenience of having a single measure is appealing for easy interpretation by environmental managers. The use of methods such as averaging of functions provides straightforward assessment of the provision of multiple functions simultaneously that could be easily added to or replicated (Maestre et al., 2012). However, the simplification of this complex information may mask important insights into what is driving individual functions. Thus, to assess the use of a multifunctionality index for prioritizing protection areas that enhance carbon functionality, an analysis balancing detail with simplification is needed (Bradford et al., 2014).

**FIGURE 1:**
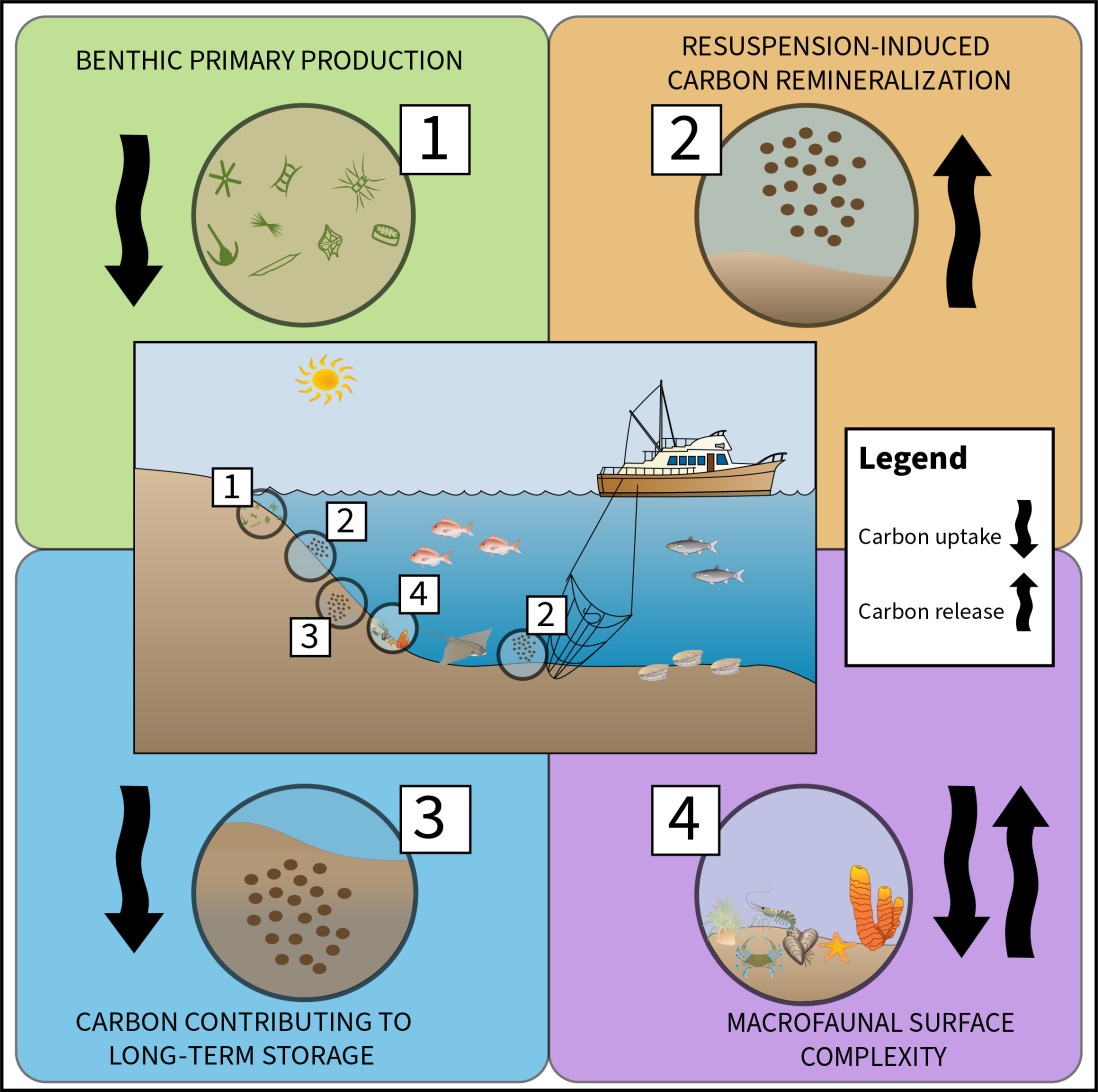
A visual representation of the functional indicators that were measured in this study, and how they impact carbon cycling in coastal soft sediment environments. The arrows depict how carbon is cycled (uptake or release) as a result of each functional process. The numbers represent the functional processes: (1) benthic primary production, (2) disturbance resulting in the resuspension of sediment, remineralizing carbon that could have otherwise been stored, (3) carbon moving to deeper long‐term stores, and (4) benthic macrofauna creating structure and features on the seafloor. Symbols courtesy of Integration and Application Network (ian.umces.edu/media‐library).

The aim of this paper was to explore the drivers of carbon cycle functionality in coastal soft sediment environments and their vulnerability to seafloor disturbance, and how this information could be incorporated into marine protection decision‐making for climate change mitigation. This was done by measuring four functional indicators of benthic carbon cycling components across environmental gradients to determine the drivers of ecosystem function, as outlined in Figure 1. The functional indicators used were chlorophyll *a* (chl *a*):phaeophytin as a surrogate for benthic primary production, surface sediment roughness as a surrogate for biodiversity, resuspension‐induced CO_2_ release to reflect the vulnerability of the seafloor to disturbance, and deep:surface organic carbon ratio as a surrogate measure for the organic carbon portion that is most likely to contribute to long‐term storage (see *Methods* for justification for these variables). The functional indicators chosen include recent advancements in the assessment of ecosystem function, in addition to established standard measurements that are often obtained in soft sediment assessments, so they have the potential to be replicated using existing or future data. We assess average multifunctionality across sites to determine whether a single measure representing multiple functions can highlight sites that provide the greatest level of multifunctionality, representing sites that should be prioritized for conservation. We hypothesize that depth would be the most important gradient for average multifunctionality, with the highest levels of functionality at shallow and deep sites. We predicted nonlinear patterns in average multifunctionality would be driven by higher primary production at shallow sites, and higher organic carbon pools in deeper sediment at deeper muddier sites (Diesing et al., 2017). Wave energy at the seabed and historical commercial dredging were predicted to influence patterns of biodiversity and resuspension‐induced CO_2_ release but were not measured as part of this study as data were not available at a relevant scale. Using insights from the functional indicators and the average multifunctionality index, we discuss how an understanding of ecosystem functions can be used to inform marine protection for outcomes that consider multiple components and complexities of the benthic carbon cycle, and the risks of continued demersal fishing in coastal environments.

## METHODS

### Data collection

**FIGURE 2:**
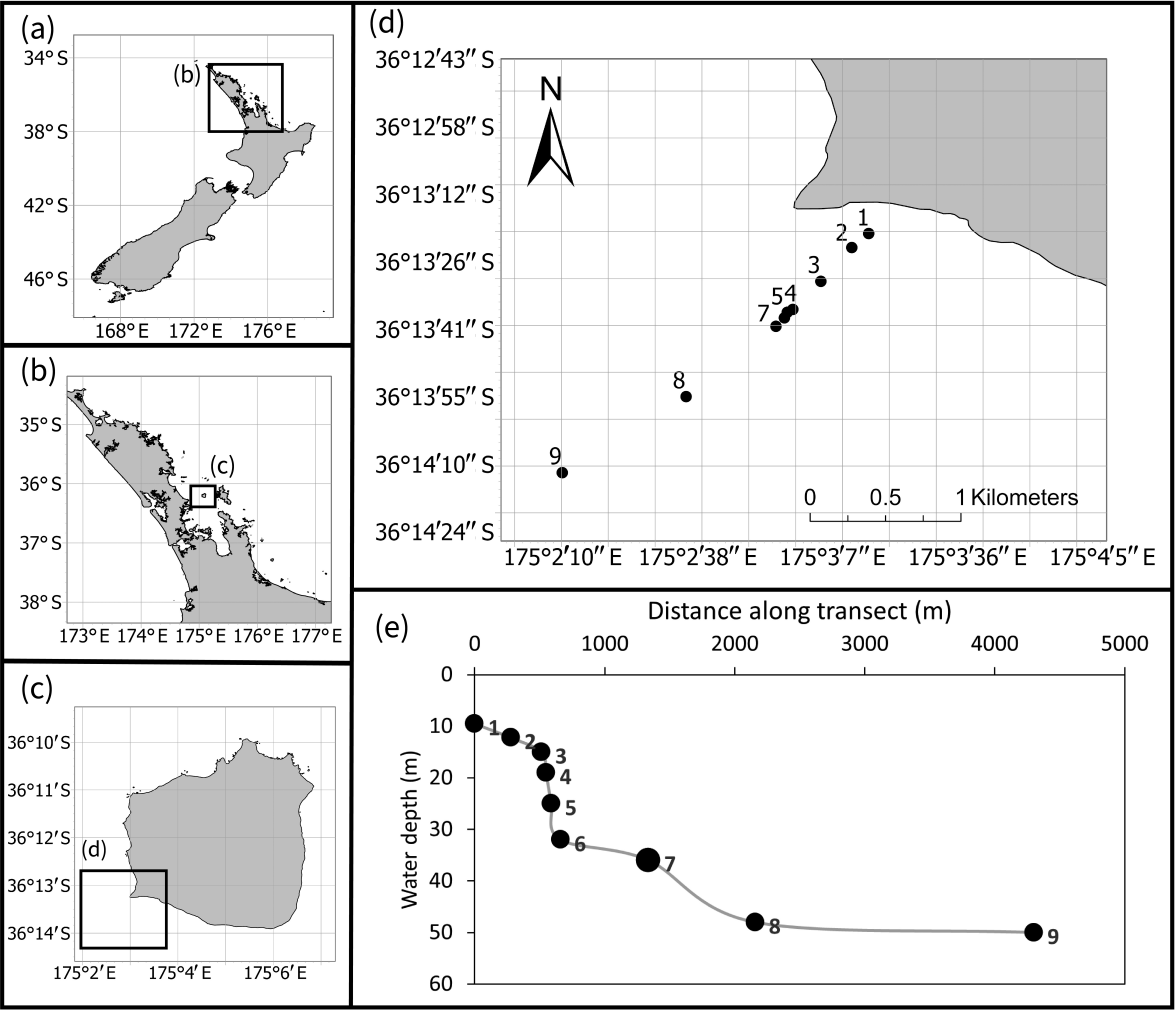
Study site in the south‐western area (c) off Hauturu (b) on the northeast coast of Aotearoa New Zealand (a), with each black circle representing a sample site along the transect (d). Panel (e) shows the depth profile along the transect.

Sampling was conducted across a transect consisting of nine sites between 4 and 11 October 2023 at Te Hauturu‐o‐Toi (hereafter “Hauturu”), an island on the northeast coast of Aotearoa New Zealand (Figure 2). The soft sediment environment surrounding Hauturu is a useful case study for this research as it represents an unfortunate and all too common example of a poorly managed shellfish fishery. Demersal fishing, by both recreational and commercial fishers, has targeted the scallop beds around Hauturu (Hauraki Gulf Forum, 2023) up until 2022, where the scallop fishery was closed due to concerns of fishery collapse (Ministry for Oceans and Fisheries, 2022). With marine protection being disproportionately located outside of coastal zones in Aotearoa New Zealand (Evans & Thrush, 2023), and worldwide (Devillers et al., 2015; Pike et al., 2024), there are global trends in shellfish bed declines following extensive demersal fishing efforts, now requiring widespread restoration efforts (Beck et al., 2011; Huang et al., 2023; Lotze et al., 2006). The location of Hauturu in the outer Hauraki Gulf provides for a variable natural disturbance regime, with intermittently high energy events (Manighetti & Carter, 1999), making it representative of other exposed, coastal sites globally.

The transect spanned a depth gradient of 10–50 m. At each site (~0.3 km^2^), three sediment cores (HAPS, KC Denmark, core diameter 14 cm) were collected and subsampled for functional indicators and environmental variables. Syringe cores (2 cm diameter) were used to subsample each core for the top 2 cm of sediment for grain size, sediment organic matter (SOM), chl‐*a* and phaeophytin content, which were kept in the dark and frozen until analysis. Syringe cores (2 cm diameter) were also used to subsample the top 3 cm of sediment, and the sediment between 8 and 10 cm for organic carbon content. These subsamples provided three replicates for each site. In addition to the subsampling for the environmental variables, six small incubation cores (acrylic tubes, inner diameter = 3.4 cm, height = 10 cm, volume = 102 mL) were subsampled at each site to assess the resuspension‐induced CO_2_ release in accordance with Bartl et al. (2024). In addition to sediment properties, a Bay Dynamics MK2 camera (Sensor: Sony IMX226, Sensor size: 9.33 mm, Pixel size: 0.00185 mm) was attached to the sediment corer that took approximately 30 s of video footage drifting at approximately 1 m from the seafloor at each site to later measure microtopographic features.

### Functional indicators

Four individual functional indicators were measured to provide insight into the four processes related to the carbon cycle, as presented in Figure 1.

Chl *a* is a key photosynthetic pigment, and its concentration in the surface sediment represents the photosynthetic standing stock, while phaeophytin (phaeo) is the non‐photosynthetic degradation product. The ratio between chl *a* and phaeo (chl *a*:phaeo) provides an indication of the quality of the photosynthetic material in the seafloor, with higher chl *a*:phaeo indicating that there is more active photosynthetic material in the sediment (Clinton et al., 2024). This was used as a proxy for microphytobenthic biomass and turnover, which is an important component of potential benthic primary production in soft sediment habitats. Chl *a* and phaeo pigments were extracted from freeze‐dried, homogenized sediment samples using 90% acetone, with concentrations determined before and after acidification using a UV–Vis spectrophotometer (Thermo Scientific, Multiskan Sky) according to Lorenzen (1967).

Variations in microtopographic surface features on the seafloor provide an indication of the physical and biological reworking of the sediment. While features like sand ripples can demonstrate physical reworking of sediment through wave action, other microtopographic features can be an indicator of biodiversity and function (Schenone, Bartl, & Thrush, 2024; Schenone, Hewitt, et al., 2024; Thrush et al., 2001). The video footage collected at each site was converted into still frames and was used to create detrended depth images, where the sediment surface was represented by positive and negative values that depict biogenically induced microtopographic features. These values were used to calculate the arithmetical mean roughness (roughness), which represents the mean of absolute deviations from the detrended surface of all frames from a site, without excluding or emphasizing any surface features. Higher surface roughness means that the sediment surface is more complex, and greater surface complexity has been linked to high biodiversity (Schenone, Hewitt, et al., 2024; Thrush et al., 2001). The measure of roughness provides insight into the benthic community at each site, where increased roughness indicates greater benthic diversity. Given the quality constraints of the camera, preprocessing of the videos included un‐distortion using OpenCV open‐source library to account for lens imperfections, optical aberrations, and environmental conditions, *Z*‐score normalization to create uniform illumination, contrast enhancement to increase the contrast between features, and color normalization using histogram matching to make the videos look more consistent. As we used a single moving camera rather than two to perceive depth, depth estimation was performed using feature matching with a Superglue network (Sarlin et al., 2020). Two sequential frames were processed using feature matching and rectification to simulate a stereo vision system (left and right) for disparity estimation (using the Stereo Matching algorithm CREStereo; Li et al., 2022), that was then converted into depth. Surface detrending and sediment microtopography characteristics were then determined using the methods outlined in Azhar et al. (2022). Full methods, including the models and code used to create the sediment microtopography characterization, can be found at https://github.com/shahrokh1106/sediment-microtopography-monocamera.

The ratio between organic carbon stored in the surface sediments (top 3 cm) and deeper in the sediment (8–10 cm) (deep:surface carbon) provides insight into the comparative organic carbon stocks between the reactive surface sediment and the stored organic carbon deeper in the sediment. These depths were chosen as organic carbon in the top 5 cm of sediment is more likely to be remineralized through natural processes (Seiter et al., 2004), and box dredges that are typically used around Hauturu dig into the top approximately 5 cm of sediment (Beentjes & Baird, 2004; Bradshaw et al., 2021), so we could identify differences in the reactive surface sediment and the sediment layer that is less likely to be disturbed. Organic carbon below the depth of surficial resuspension represents carbon that is more likely to contribute to long‐term storage; thus, a higher deep:surface carbon ratio may indicate sites with higher organic carbon storage potential. Organic carbon content was determined from freeze‐dried and homogenized sediment after acidification with HCl until effervescence had ceased, using the Vario EL Cube Elementar analyzer (Langenselbold, Germany) (Nieuwenhuize et al., 1994).

While assessing the amount of carbon that will be resuspended and remineralized during a seafloor disturbance event is difficult, we used a CO_2_ resuspension assay (developed by Bartl et al., 2024) as a simple proxy for assessing the spatial variation in CO_2_ release following resuspension. The assay incubates both disturbed (*n* = 3 per site) and undisturbed sediments (*n* = 3 per site), and the change in oxygen through time gives the sediment oxygen demand (SOD), which is used as a surrogate for organic carbon mineralization to CO_2_ (Jørgensen et al., 2022). Prior to incubation, the sediment height of all six 3.4‐cm‐diameter incubation cores was reduced to 3 cm. The undisturbed controls were carefully filled with filtered seawater and an initial O_2_ reading was taken. Cores assigned to the disturbed treatment were transferred into glass jars, filled with filtered seawater and gently and continuously inverted for 30 s to ensure full resuspension. After larger grains had settled (~30 s), the initial O_2_ reading was taken. After the initial O_2_ reading, the undisturbed cores and disturbed cores in jars were sealed ensuring no air bubbles and incubated for 5–7 h in a water bath at ambient temperature in dark conditions. At the end of the incubation period, final O_2_ readings were taken from the disturbed and undisturbed cores. The SOD for the undisturbed control (SOD_cont_) and the disturbed resuspension treatment (SOD_resus_) were calculated as:

$$\mathrm{SOD}\;\left(\text{μmol}\;{\text{m}}^{-2}\;\;h^{-1}\right)=\left(\frac{O_{2i}-O_{2f}}{V_{\mathrm{sed}}\times T\;}\right)\times h_{\mathrm{sed}},$$

where O_2*i*_ is the initial O_2_ concentration (in micromoles), O_2*f*_ is the final O_2_ concentration (in micromoles), *V*_sed_ is the bulk sediment volume (in cubic meters), *T* is the incubation time (in hours), and *h*_sed_ is the height of the sediment in the core (0.03 m). To convert SOD into organic carbon remineralization, the respiratory quotient of 0.9 for inner shelf sediments was used (Jørgensen et al., 2022). Resuspension‐induced CO_2_ production was calculated as:

$$\text{Resuspension}‐\text{induced}\;{\mathrm{CO}}_{2}\;\text{production}\;\left(\text{μmol}\;m^{-2}\;h^{-1}\right)=0.9\times \left({\mathrm{SOD}}_{\text{resus}}-{\mathrm{SOD}}_{\text{cont}}\right)$$

High values of resuspension‐induced CO_2_ represent high functionality, as greater CO_2_ release indicates more organic carbon in the sediment that could have been stored. Sites with a higher resuspension‐induced CO_2_ production are therefore more vulnerable to losing carbon cycle functionality during resuspension events.

### Multifunctionality

To assess the ability of each site to provide multiple ecosystem functions, we used an average functionality approach. For the calculation of our multifunctionality index, we needed to produce one value for each function per site. Roughness was measured at the site scale, but we averaged the replicates of chl *a*:phaeo, deep:surface carbon and resuspension‐induced CO_2_ at each site. The “multifunc” package (Byrnes et al., 2014) in R (R Core Team, 2022) was then used to standardize the individual functions on a scale of 0–1 to remove any differences based on the units of measurement, and to calculate the average multifunctionality of each site (Byrnes et al., 2014; Dooley et al., 2015; Xie et al., 2018). Missing functional indicator values were calculated as the mean of the values from the two adjacent sites on the transect, as the multifunc analysis could not handle missing data. The two missing values were at Site 7 for resuspension‐induced CO_2_ and Site 8 for roughness (i.e., <6% of the data points).

### Environmental variables

Depth, SOM, median grain size, the ratio between the reduced peak height (*S*_pk_) and the mean core roughness (*S*_k_) (*S*_pk_:*S*_k_), and the ratio between reduced valley height (*S*_vk_) and *S*_k_ (*S*_vk_:*S*_k_) were used as environmental variables to characterize the environmental variability over the transect. *S*_pk_ refers to the mean height of the peaks above the sediment surface and *S*_vk_ refers to the mean depth of the valleys below the sediment surface. *S*_k_ represents the sediment microtopography with predominant surface features removed, allowing for the determination of how dominated a site is by emergent structures (peaks) or deep holes (valleys). *S*_pk_:*S*_k_ and *S*_vk_:*S*_k_ measure the degree to which sediments are dominated by respectively tall peaks or deep valleys compared to the general variations of the sediment (Azhar et al., 2020; Schenone, Hewitt, et al., 2024). Rather than measuring the overall complexity of the sediment surface (roughness) which can be driven by the abundance and diversity of features, *S*_pk_:*S*_k_ and *S*_vk_:*S*_k_ represent the dominance of features that increase surface area. Increased surface area allows for increased solute exchange (Bianchi et al., 2021) and impacts the hydrodynamic flows in the benthic boundary layer (Moulin et al., 2007) and is thus a potentially important predictor of carbon cycling in marine soft sediments. *S*_pk_:*S*_k_ and *S*_vk_:*S*_k_ were determined using the same methods as the arithmetical mean roughness outlined earlier in this *Methods* section.

Grain size was determined using the wet sieving method following digestion of organic matter with 6% H_2_O_2_, and 5% Calgon used as a dispersant. Median grain size was calculated using the methods described in Blott and Pye (2001). SOM was determined using the loss on ignition method described in Parker (1983), where dried samples in pre‐ashed foil dishes were weighed, run in a furnace at 450°C for 4 h, and then reweighed once cooled and dried.

### Data analysis

To examine the strength of the relationships between the functional indicators and environmental predictors, single linear regressions were used to explore the relationships between each standardized response variable (chl *a*:phaeo, resuspension‐induced CO_2_, deep:surface carbon, roughness, and average multifunctionality) and each environmental predictor (depth, SOM, median grain size, *S*_pk_:*S*_k_, and *S*_vk_:*S*_k_) using the “stats” package in R (R Core Team, 2022). The environmental predictors, depth, *S*_pk_:*S*_k_, and *S*_vk_:*S*_k_, were measured at the site scale, but SOM and median grain size were averaged from site replicates to obtain one value for each site. Single linear regression models were used to identify specific relationships between the functional indicators and environmental gradients. Single linear regression models were used to minimize multicollinearity effects, to avoid overfitting due to the small sample size, and to minimize the impact of confounding variables. The residuals were visually inspected to confirm linearity. Akaike information criterion (AIC) values represent model quality by estimating the amount of variation lost by each model, where the lower the value, the better that model is at predicting the variable. The AIC values for each linear model were extracted and compared within each functional indicator (not between functional indicators) to determine which environmental variable was the best predictor for each of the functional indicators.

## RESULTS

**FIGURE 3:**
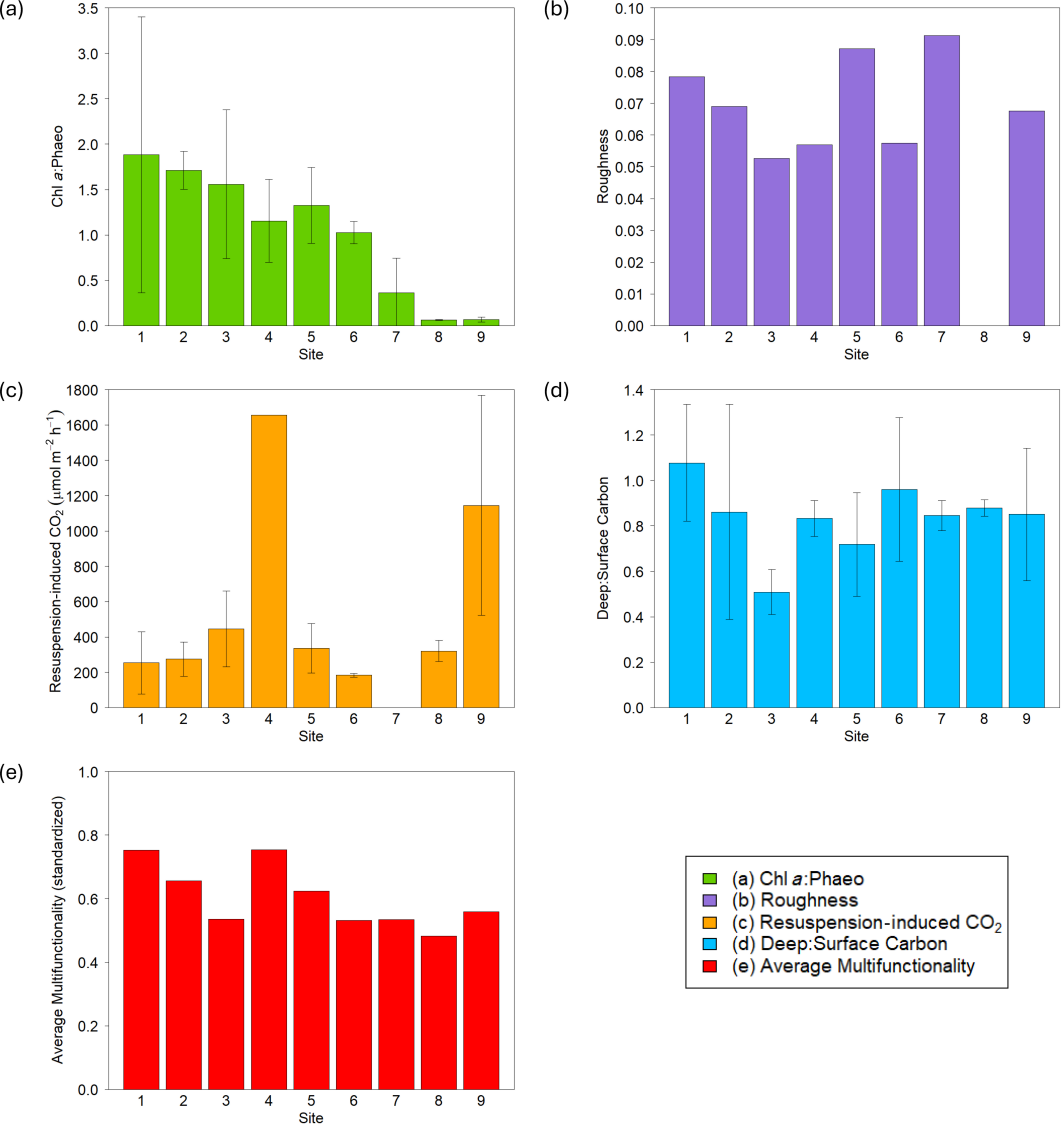
Non‐standardized indicators of the individual functions as a function of site: (a) chlorophyll *a*:phaeophytin measurements, (b) roughness, (c) resuspension‐induced CO_2_ (in micromoles per square meter per hour), (d) deep (8–10 cm):surface (less than 2 cm) organic carbon levels in the sediment, and (e) the average multifunctionality across all sites calculated by the standardized mean of the individual functional indicators. Panels (a), (c), and (d) are the means and SDs from each site (*n* = 3), except (c) Site 4 had *n* = 1. Roughness and average multifunctionality are single measurements at the site scale.

The patterns of individual functional indicators varied along the transect (Figure 3). The ratio of chl *a*:phaeo steadily decreased along the transect, before dropping off to near zero at Sites 8 and 9 (Figure 3a), suggesting a decrease in benthic primary production across the transect. Resuspension‐induced CO_2_ production ranged between 183 and 446 μmol m^−2^ h^−1^ at most sites, but with two sites (4 and 9) releasing between three and nine times more CO_2_ when resuspended (Figure 3c), which identifies these sites as being highly vulnerable to disturbance. The deep:surface carbon ratio initially decreased from 1.05 to 0.50 across the transect, then became more similar (~0.82) at the sites further along the transect (Figure 3d). The pattern of deep:surface carbon across the transect suggests that conditions influencing organic carbon storage were more consistent at sites further along the transect. There was no clear pattern of roughness across the transect (Figure 3b), indicating that benthic biodiversity is impacted by a number of variables. Average multifunctionality was highest at Sites 1 and 4, with a bimodal pattern across the transect that was a different pattern to the individual functional indicators (Figure 3e).

**FIGURE 4:**
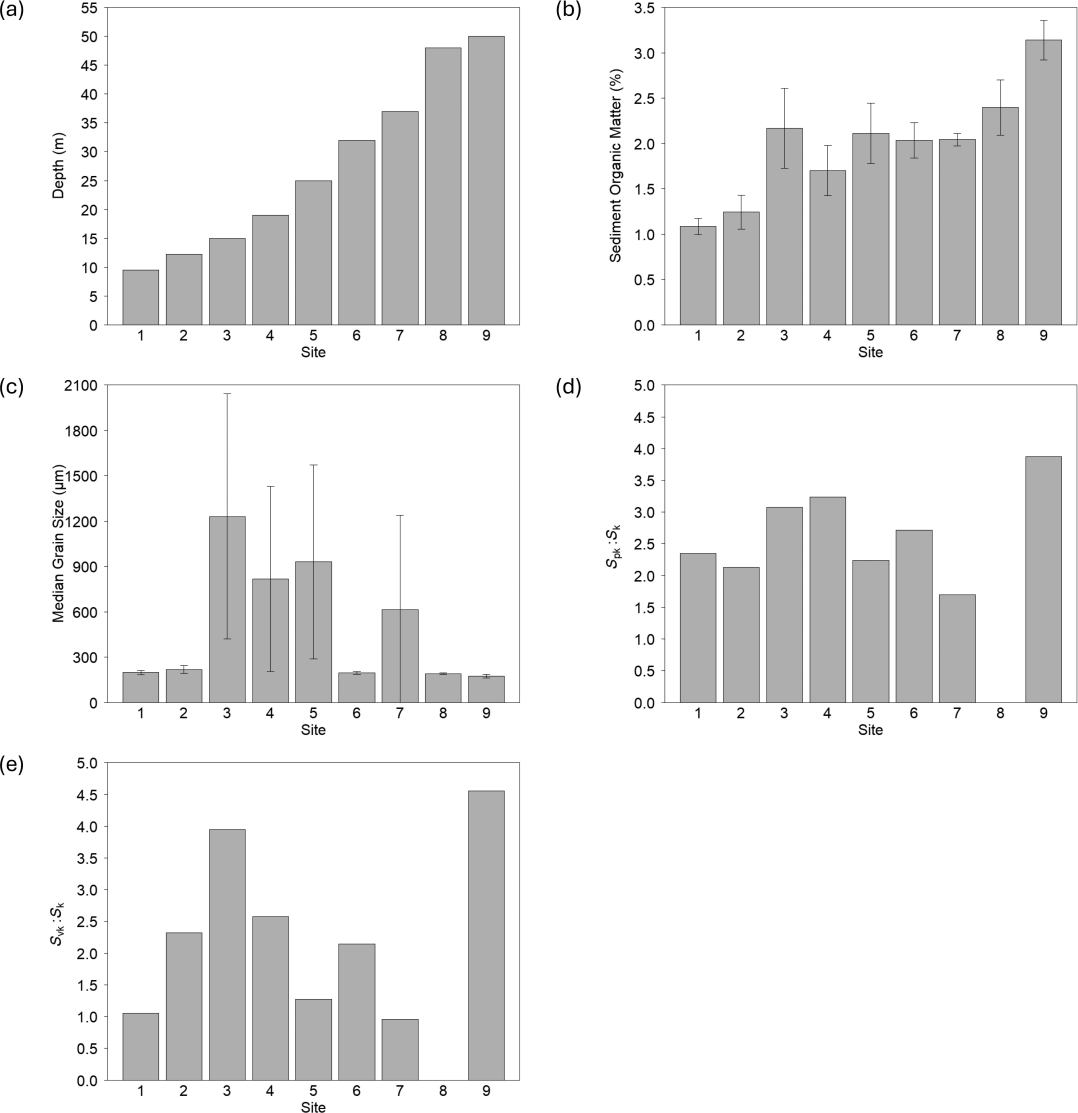
Environmental variables measured across the transect: (a) depth, (b) sediment organic matter content, (c) median grain size, (d) *S*_pk_:*S*_k_ (dominance of peaks), and (e) *S*_vk_:*S*_k_ (dominance of valleys), where the values of (a), (d), and (e) are based on a single measurement. The values of (b) and (c) are the mean and SD (*n* = 3).

SOM was the only environmental variable measured that showed an upward trend across the transect (Figure 4b). Sites with larger grain size had more within site variability, indicating that sites with larger size fractions contained a heterogeneous sediment composition. There were different patterns when comparing the dominance of peaks (*S*_pk_:*S*_k_) and valleys (*S*_vk_:*S*_k_) among the sites. Larger emergent structures were more common than the valleys (Figure 4d), but Sites 3 and 9 contained larger valleys in the sediment surface (Figure 4e).

**FIGURE 5:**
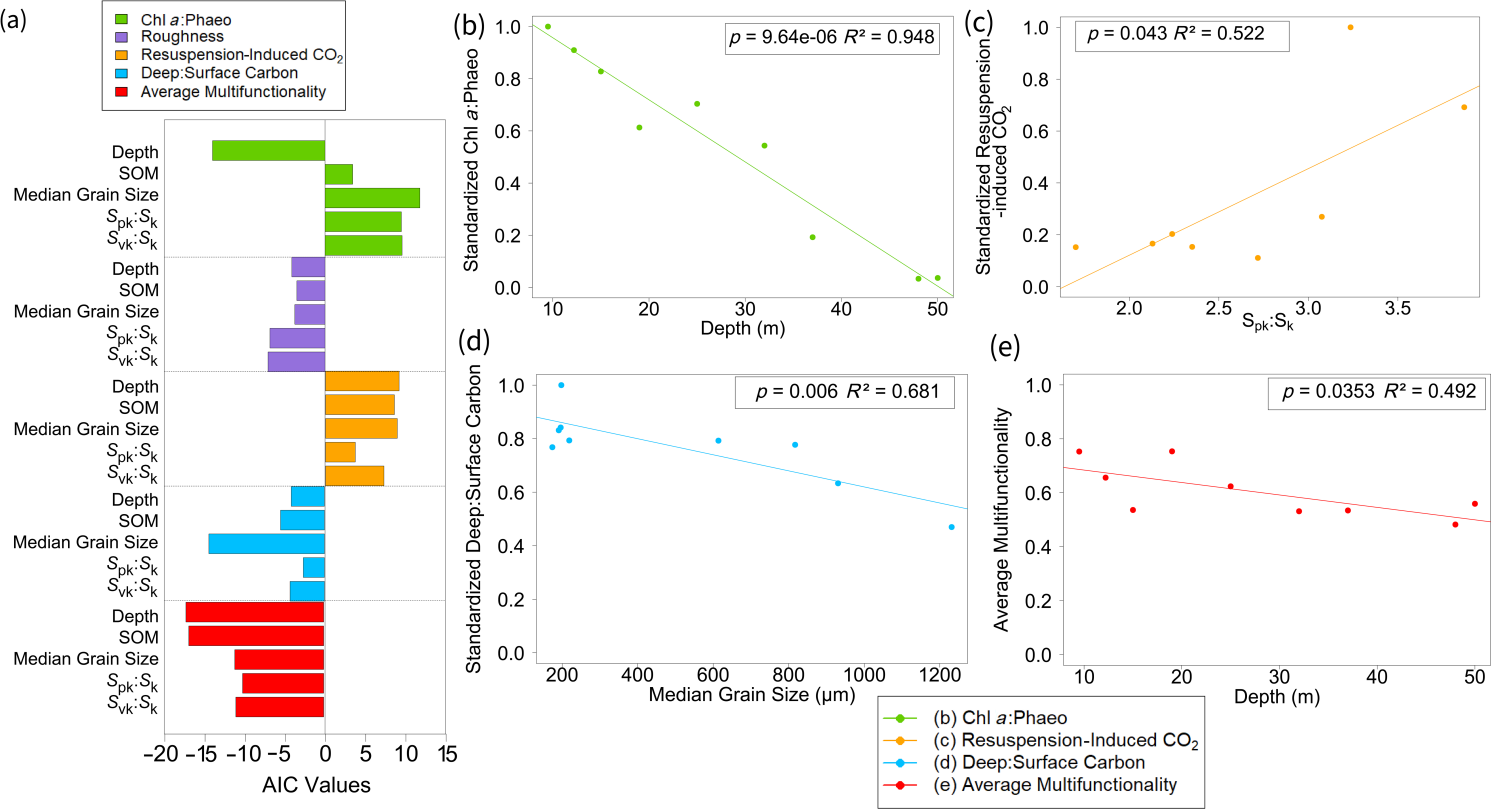
Relationships between the standardized functional indicators and the environmental variables based on single linear regressions where (a) Akaike information criterion (AIC) values show which environmental variable was the best predictor of each standardized functionality indicator. The lower (or more negative) the AIC value is, the better that model is at predicting the functional indicator. AIC values are comparable between the environmental variables for each functional indicator, but not between functional indicators. Based on the models identified by the AIC values, the linear regression between the standardized functionality indicators and the environmental variable that provided the best linear model for each functional indicator is (b) chl *a*:phaeo and depth, (c) resuspension‐induced CO_2_ release and *S*_pk_:*S*_k_, (d) deep:surface organic carbon and median grain size, and (e) average multifunctionality and depth. Roughness is not included as there were no statistically significant relationships between roughness and any of the environmental variables measured. The *p*‐values and *R* ^2^ values are above plots (b)–(e).

Each functional indicator was best predicted by different environmental variables, as demonstrated by the variation in AIC values (Figure 5). Depth was the strongest driver of chl *a*:phaeo and average multifunctionality, with SOM also being an important predictor for average multifunctionality. Deep:surface carbon ratio was best predicted by median grain size. Roughness and resuspension‐induced CO_2_ did not have a dominant predictor and were almost equally predicted by all environmental variables measured.

The functional indicators responded differently across the depth gradient, with chl *a*:phaeo having a highly statistically significant model (*p* < 0.00001). Depth explained 95% of the variation in chl *a*:phaeo, suggesting depth is a critical variable for benthic primary production (Figure 5b). There was also a statistically significant relationship between depth and average multifunctionality, but depth only explained 49% of the variation in average multifunctionality (Figure 5e). Grain size explained 68% of the variation in deep:surface carbon (*p* < 0.01), with muddy sites containing more carbon in deep sediments relative to surface sediment, compared to sandy sites (Figure 5d). There was a statistically significant relationship between *S*_pk_:*S*_k_ and resuspension‐induced CO_2_ (*p* < 0.05). Sites with a higher dominance of peaks (*S*_pk_:*S*_k_) released more CO_2_ when sediment was resuspended (Figure 5c), highlighting the vulnerability of sites with large emergent structures to sediment resuspension. Roughness did not show a significant relationship with any of the environmental variables measured.

## DISCUSSION

Understanding the drivers of carbon‐related functionality in coastal soft sediment ecosystems provides important insights for how marine protection can be used as a tool for enhancing sedimentary environments for blue carbon. Average multifunctionality displayed a bimodal pattern with depth, which did not align with our hypothesis, suggesting that there are multiple drivers of multifunctionality in this ecosystem. The individual functional indicators were best modeled by different environmental variables, highlighting the importance of incorporating complexity into assessments of multifunctionality. Given the growing importance of sedimentary environments within blue carbon frameworks (Atwood et al., 2020; Graves et al., 2022; Krabbe et al., 2022; Sala et al., 2021), it is critical that we understand the various aspects of the carbon cycle, and how this information can support evidence‐based management. A challenge for decision‐makers of marine policy is determining where to protect that maximizes multiple elements of the carbon cycle, with a need for information that is relevant to management aims (Evans & Thrush, 2023). The results of this study identify key drivers of variability in carbon cycle functionality across a transect around Hauturu, providing useful insights for identifying sites for marine protection to enhance blue carbon.

The variability in functionality between sites and the drivers of each functional indicator highlights the careful consideration that must be given to select functions and other variables that are measured when assessing multifunctionality (Epstein et al., 2022; Hölting et al., 2019). We used an averaging multifunctionality index to identify whether there was a single metric that could demonstrate functionality over environmental gradients as a possible tool to aid policymakers. However, the environmental drivers of the multifunctionality index were less clear than that of some individual functions. While the averaging approach provides a simple way of assessing multiple functions, it is not able to distinguish the drivers of individual functions (Byrnes et al., 2014). Depth was an important environmental variable for multifunctionality; however, only considering depth when aiming to maximize overall carbon functionality of the ecosystem would not adequately address sites that have higher long‐term carbon stores or are more vulnerable to resuspension‐induced CO_2_ production. The lack of insight from the multifunctionality index demonstrates the need to incorporate heterogeneity into marine protection, as a single site or gradient would not address all components of the carbon cycle.

The application of the multifunctionality index in this case may be limited due to the choice and number of functions and environmental variables chosen for this study. As there is no standardized approach to measuring multifunctionality (Byrnes et al., 2023; Diesing et al., 2017; Manning et al., 2018; Zhai et al., 2024), care should be taken on how single indices are used. A quantitative review of more than 100 multifunctionality studies across a variety of fields and study types found that the number of functions considered in the literature ranged from 3 to 27 (Hölting et al., 2019), which would impact the overall multifunctionality results. While it may not be as informative in selecting sites for marine protection in this study, Villnäs et al. (2013) found that multifunctionality approaches may be able to predict decreases in functionality earlier than individual functions, so this approach may be better suited to identifying temporal changes. Using measures of multifunctionality over temporal scales could make multifunctionality indices useful in monitoring the success of marine protection in enhancing functionality. For the identification of areas for marine protection, however, individual functional indicators should be used over multifunctionality indices.

The lack of clear patterns and drivers in roughness in this study could be a result of the disturbance history at Hauturu. While there was no data available at the time of completing this study at an applicable scale to model the relationship between fishing disturbance and roughness as an indicator of biodiversity, the scallop fishery in this area was closed the year prior to sampling due to concerns of fishery collapse (Ministry for Oceans and Fisheries, 2022), indicating large‐scale degradation of the benthic community. Hauturu is characterized by small, opportunistic taxa (Evans, 2025b) that would be creating less surface roughness, making differences in biodiversity between sites harder to detect using microtopographic features as a proxy for biodiversity. The implementation of strict no‐take marine protection would reduce the widespread intensity of disturbance around Hauturu, allowing diversity to recover. Assessing roughness following recovery would provide an opportunity for further insight into the role of biodiversity in carbon cycle functioning, as at sites with increased diversity, we could expect the standing stock and burial of carbon to increase (Legge et al., 2020; Middelburg, 2018). It will therefore be important to consider the role of anthropogenic disturbance not only on diversity but also on the overall functioning of blue carbon ecosystems.

Grain size plays an important role in how demersal fishing impacts carbon cycling functionality. Muddy sites store more carbon than sandier sites, which can also make them more vulnerable to the impacts of demersal fishing as there is more organic carbon that can be resuspended when disturbed (Sciberras et al., 2016). Methods like dredging are normally targeted at specific sites of known species distributions, meaning that the same sites can be dredged on multiple occasions, with each fishing event uncovering carbon that would have otherwise been stored (Atwood et al., 2023). For these muddy sites that are frequently fished, this results in lower sediment carbon content compared to sites with lower levels of disturbance (Hale et al., 2017). While natural disturbances like wave energy at the seabed can also resuspend sediment, demersal fishing often occurs at sites below the wave base impacting areas of the seafloor that could otherwise remain undisturbed (Oberle, Storlazzi, & Hanebuth, 2016). As indicated by the strong decrease in chl *a*:phaeo with depth, benthic primary production in this environment was limited by light (Epstein et al., 2022), so reductions in light levels with increased turbidity caused by demersal fishing could decrease the seafloor's role in carbon capture. Not only does the resuspension of sediment release CO_2_ and reduce possible carbon storage, but as the fine fractions are winnowed away, the increased grain size can reduce the capacity of the seafloor to store carbon in the future. To enhance carbon capture and storage around Hauturu, marine protection should cover both shallow sites to conserve carbon capture by benthic primary production and muddy sites to conserve organic carbon storage.

In addition to enhancing carbon capture and storage, minimizing CO_2_ release is important in the face of climate change (Luisetti et al., 2019). By simulating disturbance events, we were able to assess the variability in resuspension‐induced CO_2_ release across sites (Bartl et al., 2024). The sites most vulnerable were those with larger emergent surface features, suggesting that not only are large fauna most vulnerable to demersal fishing (Dayton et al., 1995; Thrush et al., 2006; Thrush & Dayton, 2002), but so are the sedimentary habitats that they live within. While macrofauna were not included within the resuspension assays, this result demonstrates the importance of fauna in mediating sedimentary processes linked to carbon storage. For example, structures on the seafloor can impact hydrodynamics and accumulate organic matter through a sheltering effect (Moulin et al., 2007), suggesting that the dominance of sediment surface peaks can have positive impacts on carbon cycling, provided that sediment is not resuspended. Epifauna on the seafloor are also important for supporting other diversity, particularly juveniles (Bradshaw et al., 2003). Marine protection could therefore protect sites with benthic fauna that create large emergent structures to avoid releasing a greater amount of CO_2_, which can help to address both biodiversity and climate change concerns (Roberts et al., 2020; Shin et al., 2022).

Efforts are increasing to quantify the functionality of the seafloor to store and release carbon to support global policy discussions (Epstein et al., 2022; Krabbe et al., 2022; Luisetti et al., 2019; Sala et al., 2021; Sciberras et al., 2016). To make these efforts meaningful, we need to push for increased marine protection globally (Roberts et al., 2020). For countries such as Aotearoa New Zealand that have large EEZ's and allow demersal fishing within coastal areas, implementing marine protection that prohibits bottom contact fishing in strategic locations could play an important, and globally relevant, role in protecting carbon stocks (Sala et al., 2021). This study provides important insights into the variability of functionality across environmental gradients in coastal soft sediment environments, demonstrating that disturbing the seafloor, through activities such as demersal fishing, negatively impacts carbon cycle functionality in coastal soft sediment ecosystems, which has implications for increasing ocean acidification and exacerbating climate change (Epstein et al., 2022; Sala & Knowlton, 2006; Sciberras et al., 2016; Tiano et al., 2019). We encourage the adoption of using either measures of function or functional indicators, and the development of measures of multifunctionality, more widely across coastal soft sediment ecosystems to support informed management. By accounting for environmental variability and complexity in marine protection, we have the opportunity to enhance carbon capture and storage, as well as manage the vulnerability of sites to resuspension‐induced CO_2_ release.

## AUTHOR CONTRIBUTIONS

Tegan Evans designed the study, conducted the data collection and data analysis, and wrote the manuscript. Ines Bartl designed and conducted the resuspension assay, provided support with data collection, analysis, and manuscript editing. Shahrokh Heidari processed the images to generate microtopographic surface features. Rebecca V. Gladstone‐Gallagher provided support with analysis and manuscript editing. Stefano Schenone provided support with data collection, analysis, and manuscript editing. Patrice Delmas provided support on the generating microtopographic surface features. Simon F. Thrush provided support with study design, analysis, and manuscript editing.

## FUNDING INFORMATION

This research was funded by the Oceans of Change project (The Auckland Foundation) and MBIE UOAX2307 and was supported by Our Seas Our Future.

## CONFLICT OF INTEREST STATEMENT

The authors declare no conflicts of interest.

## Data Availability

Data (Evans, 2025a) are available in Figshare at https://doi.org/10.17608/k6.auckland.28701986.
